# Historical land use has long-term effects on microbial community assembly processes in forest soils

**DOI:** 10.1038/s43705-021-00051-x

**Published:** 2021-09-10

**Authors:** Ernest D. Osburn, Frank O. Aylward, J. E. Barrett

**Affiliations:** Department of Biological Sciences, Virginia Tech, Blacksburg, VA USA

**Keywords:** Microbial ecology, Forest ecology

## Abstract

Land use change has long-term effects on the structure of soil microbial communities, but the specific community assembly processes underlying these effects have not been identified. To investigate effects of historical land use on microbial community assembly, we sampled soils from several currently forested watersheds representing different historical land management regimes (e.g., undisturbed reference, logged, converted to agriculture). We characterized bacterial and fungal communities using amplicon sequencing and used a null model approach to quantify the relative importance of selection, dispersal, and drift processes on bacterial and fungal community assembly. We found that bacterial communities were structured by both selection and neutral (i.e., dispersal and drift) processes, while fungal communities were structured primarily by neutral processes. For both bacterial and fungal communities, selection was more important in historically disturbed soils compared with adjacent undisturbed sites, while dispersal processes were more important in undisturbed soils. Variation partitioning identified the drivers of selection to be changes in vegetation communities and soil properties (i.e., soil N availability) that occur following forest disturbance. Overall, this study casts new light on the effects of historical land use on soil microbial communities by identifying specific environmental factors that drive changes in community assembly.

Soil microbial communities play key roles in terrestrial ecosystems, facilitating essentially all ecosystem processes [1]. However, soil microbial communities globally are threatened by land use change, which has modified ~75% of earth’s ice-free land area [2] and has had wide-ranging effects on soil microbial community structure [3, 4] and ecosystem functions [5, 6]. These changes in microbial communities are linked to differences in community assembly processes (i.e., selection, dispersal, drift) among land uses [7], which, in turn, are driven by specific environmental factors (e.g., soil properties) that influence soil microorganisms [3, 4]. For example, changes in soil pH exert strong selective pressure on microorganisms [8] and pH differences among land uses influence the relative importance of different assembly processes in structuring soil microbial communities [9].

In addition to effects of present land use, currently unmanaged ecosystems with different historical management regimes may also host distinct microbial communities [10]. For example, historical forest management (e.g., logging, agriculture conversion) influences soil microbial communities for several decades after management activities have ceased and forest recovery has occurred [10]. These influences include increased bacterial diversity and increased abundance of specific microbial functional groups (e.g., nitrifiers and r-selected bacteria) in previously managed sites [10]. However, the community assembly processes and related environmental drivers that underlie these legacy effects of historical land use have not been identified. We predicted that changes in key soil properties following historical management (e.g., increased pH and inorganic N) would serve as an environmental filter, thereby increasing the importance of homogenous selection in structuring bacterial and fungal communities in historically managed soils.

To investigate microbial community assembly across historical land uses, we sampled soils from eight forested watersheds at the Coweeta Hydrologic Lab in North Carolina, USA. Four of the watersheds were disturbed ~4–8 decades previously by management activities including clear-cutting, cable-logging, conversion to pine monoculture, and pasture conversion (Table S1). Adjacent to each historically disturbed forest is a “reference” watershed that has remained undisturbed for ~100 years (Fig. S1). Within each watershed, we established six plots, surveyed vegetation, and sampled 10 cm depth soils from each plot. Sampling took place in June 2018, at the height of the growing season. We measured soil properties (Table S2) and characterized bacterial and fungal communities by amplicon sequencing of the 16S rRNA and the ITS1 regions, respectively. All sampling and sequencing methods were previously described [10]. We aligned 16S sequences to SILVA [11] and aligned ITS sequences using PASTA [12], which is appropriate for regions with high sequence length variability such as ITS [13]. We then used FastTree2 [14] to construct maximum-likelihood phylogenetic trees for bacteria and fungi (Figs. S2, S3). Due to potential issues with ITS sequence alignment, we repeated all analyses using a fungal tree based on the UNITE taxonomy for our fungal OTUs, which produced nearly identical results compared with the sequence alignment approach (Figs. S4, S5).

We quantified the relative importance of different community assembly processes using the null model approach described by Stegen et al [15], which assumes that closely related taxa are also ecologically similar. We confirmed this assumption for our data using the mantel correlogram method [15] (Figs. S6 and S7). The null model method distinguishes between selection and neutral processes by calculation of standardized phylogenetic turnover between communities (i.e., βNTI). Phylogenetic turnover less than or greater than null expectations indicates the homogeneous or variable selection, respectively. Turnover that does not differ from null expectations indicates neutral processes. Standardized compositional turnover (i.e., RC_Bray_) then distinguishes between specific neutral processes, where compositional turnover less than or greater than null expectations indicates homogenizing dispersal or dispersal limitation + drift, respectively. Turnover not differing from null indicates drift alone. We determined differences between bacterial and fungal βNTI distributions using a Kruskal–Wallis test and determined effects of historical disturbance on the proportions of specific processes using *Z*-tests. We investigated potential drivers of selection (i.e., vegetation and soil properties) by conducting variation partitioning on βNTI matrices, similar to the approach of Fillinger et al [16]. All methodological details regarding sampling, lab methods, and statistical analyses are provided in the Supplementary Information.

For both bacteria and fungi, communities were structured primarily by neutral processes, with stochastic drift particularly important in bacterial communities (Fig. 1). However, bacteria exhibited significantly lower βNTI than fungi (Fig. 1A), indicating greater importance of homogenous selection in structuring bacterial communities and greater importance of neutral processes in structuring fungal communities, which has also been previously reported [13, 17]. Specifically, fungal communities were primarily structured by dispersal limitation (Fig. 1C), possibly attributed to the large proportion of dispersal-limited mycorrhizal taxa (~30% of sequences) present in these soils (Fig. S8) [18]. The importance of homogeneous selection in structuring bacterial communities is consistent with the strong and well-known relationships between bacterial communities and specific soil properties (e.g., pH, N availability) [3, 4]. These domain-level differences in community assembly processes likely reflect fundamental differences in bacterial and fungal life history strategies, e.g., differences in growth, dispersal, and/or dormancy methods.

**Fig. 1:**
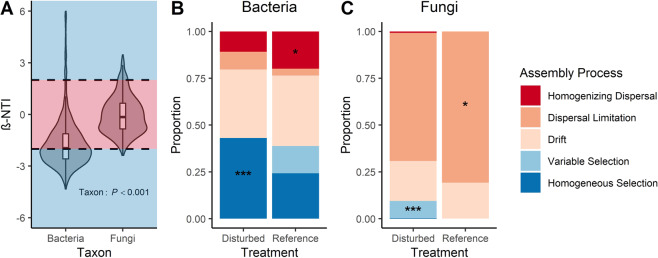
Community assembly processes for bacterial and fungal communities in ‘reference’ and historically ‘disturbed’ forest soils. In (**A**), βNTI distributions for bacteria and fungi are shown, while (**B**) and (**C**) show proportions of community assembly processes between historical land uses for bacteria and fungi, respectively. In all panels, shades of blue represent selection processes while shades of red represent neutral processes. In (**A**), phylogenetic turnover that is less than null expectations (i.e., βNTI < −2) indicates homogenous selection, phylogenetic turnover that is greater than null expectations (i.e., βNTI > 2) indicates variable selection, and phylogenetic turnover that does not vary from null expectations (|βNTI | < 2) indicates neutral processes. In (**A**) the *P* value is from a Kruskal–Wallis test. In (**B**) and (**C**), asterisks represent statistically greater proportions (*Z*-tests) at the following significance levels: *P < 0.05, ***P < 0.001. In (**B**), selection overall (i.e., homogeneous + variable) is greater in disturbed soil communities (*P* = 0.046).

Bacterial and fungal communities in historically disturbed sites were more structured by selection than reference communities (Fig. 1B, C). Variation partitioning identified the drivers of selection to be specific vegetation and soil properties (i.e., N availability, Table 1). Vegetation accounted for particularly large proportions of variation in both bacterial and fungal βNTI (Table 1), indicating that changes in vegetation and changes in soil N that are dependent on vegetation account for the greater selective pressures in disturbed sites. For example, the greater influence of homogeneous selection on disturbed soil bacterial communities (Fig. 1B) likely reflects consistent bacterial responses to increased N availability, e.g., increased abundance of r-selected and nitrifying taxa [4]. These increases in N availability, in turn, are likely linked to vegetation changes following disturbance, e.g., increased abundance of tulip poplar (*L. tulipifera*) and the historical dominance of N-fixing *Robinia pseudoacacia* in the disturbed sites (Table 1, S1) [19]. The greater influence of variable selection on disturbed soil fungal communities (Fig. 1C) suggests the existence of distinct fungal niches in different disturbed forests due to current vegetation differences, e.g., *Pinus* monoculture vs. hardwood forest (Table 1, S1). Though other studies have reported the predominance of neutral processes in recently disturbed ecosystems [17, 20], we show that selection becomes more important later in ecosystem succession [21].

**Table 1:** Partitions of variation in βNTI accounted for by soil variables and vegetation communities.

Taxon	Partition	Adj. *R*^2^	*P* value	Significant variables
Bacteria	Soil	0.17	0.004	Total N, Extractable N, Extractable C:N
	Vegetation	0.32	0.001	*Q. rubra, P. strobus, B. lenta, L. tuilipifera*
	Soil + Vegetation	0.45	0.001	
	Soil | Vegetation	0.05	0.003	Total N, Extractable C:N
	Vegetation | Soil	0.14	0.001	*Q. rubra, P. strobus, B. lenta*
	Soil ∩ Vegetation	0.19	−	
	Residuals	0.55	−	
Fungi	Soil	0.24	0.001	Total N, Microbial Biomass C
	Vegetation	0.33	0.001	*R. maximum, P. strobus, L. tulipifera, Carya spp*.
	Soil + Vegetation	0.38	0.001	
	Soil | Vegatation	0.05	0.005	Total N
	Vegetation | Soil	0.14	0.001	*R. maximum, P. strobus, L. tulipifera*
	Soil ∩ Vegetation	0.19	−	
	Residuals	0.62	−	

The significance of each partition was tested using distance-based redundancy analysis (dbRDA). Note that the significance of Soil ∩ Vegetation cannot be tested. The significance of individual variables within each variation partition was determined using permutation tests (anova.cca function, vegan package) following dbRDA. For vegetation species, genus abbreviations are as follows: *Q* *Quercus*, *P* *Pinus*, *B* *Betula*, *L* *Liriodendron*, *R* *Rhododendron*.

Dispersal processes were more important in structuring bacterial and fungal communities in reference soils than in disturbed soils (Fig. 1B, C). Greater homogenizing dispersal in reference soil bacteria (Fig. 1B) may be attributed to greater age and stability of the reference forests. In fungi, greater dispersal limitation in reference soils (Fig. 1C) may again reflect vegetation differences, which promote greater abundance of dispersal-limited mycorrhizae in reference sites vs. disturbed sites (36 and 25% of sequences, respectively, Fig. S8) [18].

Overall, our results reveal clear legacies of historical land use on soil microbial community assembly; contrary to what has been shown for recently disturbed systems, microbial communities in historically disturbed sites can be more strongly shaped by selection than their undisturbed counterparts. However, it is possible that these patterns are specific to our soils within temperate forests of the eastern US, which should be assessed by future work. Regardless, the differences in community assembly we report are likely to influence ecosystem recovery following forest disturbance. For example, ecosystem processes associated with specific fungal taxa may recover slowly or not at all following forest disturbance, as these taxa will not be quickly recruited from undisturbed sites via dispersal (Fig. 1C). In general, this study casts new light on the mechanisms that drive changes in microbial communities following forest disturbance, with implications for the key ecosystem processes that these communities facilitate.

## Supplementary information

Supplementary information

## Data Availability

All data and R scripts used in statistical analyses are available at the author’s GitHub repository at the following URL: https://github.com/eosburn/Coweeta-Microbes/tree/master/Community_Assembly
