# Supplementary material for: Historical land use has long-term effects on microbial community assembly processes in forest soils
Source: ISME Commun. 2021 Sep 10;1:48. doi: 10.1038/s43705-021-00051-x (PMC9723674; doi:10.1038/s43705-021-00051-x)
Supplement: Supplementary file 1 — Supplementary information [file 43705_2021_51_MOESM1_ESM.docx]

**Supplementary** **Information**: Historical land use has long-term effects on microbial community assembly processes in forest soils

**Supplemental Methods**

*Site Description and Soil Sampling*

We conducted this study at the Coweeta Hydrologic Lab, a USDA Forest Service experimental forest in the Appalachian Mountains of western North Carolina, USA. Within the Coweeta basin, we sampled soils from eight forested watersheds (Fig. S1). Four of the watersheds experienced whole-watershed disturbances ~4–8 decades previously as a result of forest management experiments conducted by the Forest Service (Table S1). Disturbances included clear-cutting, cable logging, conversion to pasture, and conversion to pine monoculture (Table S1, Fig. S1). Adjacent to each historically disturbed watershed is a reference watershed that has not been manipulated by the Forest Service (Fig. S1). Within each watershed, we sampled soils from six 4 m x 4 m plots evenly spaced along a 200 m transect located 5 m upslope from the stream channel. Within each plot, we surveyed all woody vegetation by recording the abundance of each woody species within each plot. We also sampled five 10 cm depth mineral soil (A horizon) cores (from four plot corners and plot center), composited cores by plot and passed composite soil samples through a 4 mm sieve. Sampling took place in June 2018, at the height of the growing season. Subsamples were stored at 4 ℃ prior to measurement of soil properties or -20 ℃ prior to DNA extraction. We measured soil moisture, pH, NO_3_^-^, NH_4_^+^, total dissolved N (TDN), extractable organic C (DOC), total C, total N, and microbial biomass C and N according to previously described methods [1] and these data are provided in Table S2. NO_3_^-^ and NH_4_^+^ were determined in 2M KCl extracts (1:5 soil:solution) while TDN, DOC, microbial biomass C, and microbial biomass N were determined in 0.5M K_2_SO_4_ extracts (1:5 soil:solution). No correction factor was applied to microbial biomass values.

*DNA extraction, PCR, and Amplicon Sequencing*

We extracted DNA from ~0.25 g soil using a Qiagen DNeasy PowerSoil kit (Qiagen, Valencia, CA, USA) we quantified extracts using a Qubit 2.0 fluorometer (Thermo Fisher Inc, Waltham, MA, USA). We characterized bacterial and fungal communities via amplicon sequencing of the V4 region of the 16S rRNA gene and the ITS1 region, respectively. We amplified 16S using the 515f/806r primer pair [2, 3] and ITS using the ITS1f/2 primer pair [4]. PCR reactions and library preparation for sequencing were performed according to previously described methods [1]. Briefly, samples were amplified in triplicate and PCR reactions contained 10 µl Thermo Fisher Platinum II Hot Start PCR Master Mix (Thermo Fisher Inc, Waltham, MA, USA), 1 µl undiluted DNA template (~5-20 ng DNA), 0.2 µM forward and reverse primer, and nuclease-free H_2_O to 25 µl. In addition, we amplified negative controls for each barcoded PCR primer to detect possible contamination. Thermal cycling conditions for 16S amplification were 2 min at 94°C followed by 35 cycles of 45 s at 94°C, 60 s at 50°C and 90 s at 72°C, with a 10 min final extension at 72°C while conditions for ITS were 2 min at 94°C followed by 35 cycles of 30 s at 94°C, 30 s at 52°C and 30 s at 68°C, with a 10 min final extension at 68°C. After amplification, we pooled triplicate PCR amplicons, visualized amplicons and negative controls on an agarose gel, and purified amplicons using the Qiagen QIAquick PCR Purification Kit (Qiagen, Valencia, CA, USA). We then pooled 16S and ITS amplicons separately in equimolar ratios and sequenced amplicons on the Illumina MiSeq platform using 250 bp paired-end reads. Raw sequence reads were deposited in the NCBI BioProject database under accession number PRJNA548911.

*Bioinformatic Processing*

Due to poor quality scores for the reverse reads, only forward reads were analyzed, and 16S and ITS sequences were trimmed to 248 and 232 base pairs, respectively. Sequence reads were processed in QIIME2 [5] using the DADA2 pipeline [6]. After DADA2 processing, we clustered sequences into 97% OTUs using VSEARCH [7] and removed OTUs only appearing in one sample. After processing, we retained 3,757,216 16S sequences and 699,218 ITS sequences for further analysis. We then randomly sampled 21,590 16S sequences and 3,310 ITS sequences from each sample to account for differences in sequencing depth among samples. After random sampling, we retained 3,551 16S OTUs and 1,197 ITS OTUs. We assigned taxonomy to OTUs using a naïve-bayes classifier [8] trained on the SILVA (version 138.1) and UNITE (version 8.2) databases for 16S and ITS, respectively [9, 10].

Prior to statistical analyses, we constructed bacterial and fungal phylogenetic trees following a previously described method [11]. Briefly, for bacteria, we aligned 16S sequences to the SILVA database [12] and constructed a maximum-likelihood tree using the aligned sequences with FastTree2 [13]. For fungi, we conducted multiple sequence alignment using PASTA [14]. The PASTA divide-and-conquer algorithm first divides sequences into smaller subsets based on similarity, aligns each subset separately, and then merges the subset alignments into a final, global alignment. This method is effective for aligning regions with high sequence length variability such as ITS. The aligned ITS sequences were then used to create a maximum-likelihood tree using FastTree2 [13]. Maximum-likelihood trees for bacteria and fungi are provided on Figs. S2 and S3, respectively. Though the ITS region is not considered to be alignable for tree-wide analyses, ITS is considered reliable for family or genus level analyses [15], i.e., near the tips of the phylogeny. Therefore, because our community assembly analyses reflect patterns at the tips of the phylogeny (i.e., βNTI values, see below), our ITS results are likely reliable. Nevertheless, for verification purposes, we repeated all analyses using a fungal phylogenetic tree derived from the UNITE taxonomy for our fungal OTUs using the *taxonomy_to_tree.pl* script from Tedersoo et al. [15]. The taxonomy-based tree and the ITS sequence alignment tree produced nearly identical results for all community assembly analyses (Figs. S4, S5). Alternative data analysis scripts for each of the fungal trees is available at the author’s github repository at the following URL: https://github.com/eosburn/Coweeta-Microbes/tree/master/Community_Assembly

*Data Analysis*

Statistical analyses were performed in R [16] using the vegan and iCAMP packages [17, 18]. To quantify community assembly processes in bacterial and fungal communities across land uses, we used the null model approach described by Stegen et al. [19]. This method rests on the assumption that closely related taxa are also ecologically similar. We tested this assumption with an approach used by multiple previous studies [11, 19, 20], beginning with determination of optimal environmental conditions for each OTU by calculating relative abundance-weighted mean values for each environmental variable (i.e., soil variables) for each OTU. Then, we calculated a between-OTU Euclidean distance matrix with respect to these environmental optima and used a Mantel correlogram (‘mantel.correlog’ function, vegan package) to relate between-OTU environmental optima to between-OTU phylogenetic distances. We determined statistical significance of relationships by permuting the distance matrix 1000 times with *P* values corrected using the Bonferroni method. Similar to many prior studies [11, 19, 20], we detected phylogenetic signal (i.e., significant positive correlations) only at short phylogenetic distances for both bacteria (Fig. S6) and fungi (Fig. S7), thus justifying use of the Stegen et al. method.

The Stegen et al. null model approach is a two-part method, beginning with calculating standardized effect sizes of β-mean nearest taxon distance (i.e., βNTI) between communities. We determined βNTI using a null distribution of β-mean nearest taxon distance values where taxa are randomly shuffled across the phylogeny with 1000 permutations. A βNTI value < -2 indicates less phylogenetic distance between communities than expected by chance, indicating selection of phylogenetically (and ecologically) similar species in both communities (referred to as ‘homogeneous selection’). A βNTI value > 2 indicates greater phylogenetic distance between communities than expected by chance, indicating selection of ecologically dissimilar species in the two communities (referred to as ‘variable’ selection). Where |βNTI| < 2, phylogenetic distance between samples is not different from null expectations, indicating neutral assembly processes account for differences (or lack thereof) between the communities. Because phylogeny is assumed to be irrelevant to neutral processes, in part two of the method, only compositional information (i.e., presence/absence and relative abundance of taxa) is used to quantify contributions of specific neutral processes. In this step, the RC_Bray_ index is calculated, which is a standardized compositional turnover metric, i.e., a Raup-Crick null model is used to standardize Bray-Curtis dissimilarities between communities. RC_Bray_ is standardized between -1 and 1, with values < -0.95 indicating communities that are more compositionally similar than expected by chance, which is interpreted as homogenizing dispersal being responsible for the high compositional similarity of the communities. RC_Bray_ > 0.95 indicates two communities that are more compositionally different than expected by chance, which is interpreted as dispersal limitation + drift being responsible for the large compositional dissimilarity between the communities. |RC_Bray_| < 0.95 indicates compositional difference between communities that does not deviate from null expectations, which is interpreted as drift acting alone. βNTI and RC_Bray_ indices were calculated using the ‘qpen’ function in the iCAMP package. We tested for differences between taxa (i.e., bacteria vs. fungi) in βNTI distributions using a Kruskal-Wallis test. Within taxa, we tested for effects of historical disturbance on the proportions of specific assembly processes using Z-tests.

We assessed potential environmental drivers of selection by conducting variation partitioning on the βNTI matrices for bacteria and fungi (‘varpart’ function, vegan package), similar to the approach of Fillinger et al. [21]. We considered soil properties, vegetation communities, and spatial factors as candidate driver variables. Spatial factors were represented by principle coordinates of neighborhood matrix (PCNM) scores for each field plot (‘pcnm’ function, vegan package). We selected specific variables for variation partitioning by constructing a distance-based redundancy analysis (dbRDA) model (‘capscale’ function, vegan package) for each variable category containing the full set of variables in that category and then used the ‘ordistep’ model selection procedure (vegan package) to select the best-supported dbRDA model for each category. Only variables in the best-supported dbRDA model were included in the final variation partitioning analysis. We determined statistical significance of individual driver variables using a permutation test (‘anova.cca’ function, vegan package). Prior to analyses, we scaled all soil variables between 0 and 1 and Hellinger-transformed the vegetation species abundance data. We omitted spatial variables from the final variation partitioning analyses because they did not independently account for any variation in βNTI for either bacteria or fungi. We determined statistical significance of each variance partition using dbRDA. Because dbRDA requires positive distance values, we scaled βNTI values to range between 0 and 1 prior to analysis.

Table S1: Detailed watershed information for all eight watersheds sampled in this study.

| Watershed Pair | Watershed Number | Treatment | Aspect | Elevation (m) | Area (ha) | Dominant Woody Vegetation |
| --- | --- | --- | --- | --- | --- | --- |
| Cable Logged | WS2 | Undisturbed since 1923 | SSE | 709 – 1004 | 12 | *Rhododendron maximum, Quercus spp., Betula spp.* |
|  | WS7 | Commercially clear cut and cable logged in 1977 | S | 772 – 1077 | 59 | *Rhododendron maximum, Quercus montana., Acer rubrum, Liriodendron tulipifera* |
| Pasture Conversion | WS14 | Undisturbed since 1923 | NW | 707 – 992 | 61 | *Rhododendron maximum, Liriodendron tulipifera, Betula spp.* |
|  | WS6 | Clearcut in 1958, soil scarified, planted to grass, limed and fertilized in 1959, fertilized again in 1965, grass herbicided in 1966 and 1967 | NW | 696 – 905 | 9 | *Acer rubrum, Liriodendron tulipifera* |
| Pine Conversion | WS18 | Undisturbed since 1923 | NW | 726 – 993 | 13 | *Rhododendron maximum, Acer rubrum, Betula spp.* |
|  | WS17 | All woody vegetation cut annually 1940 - 1955, white pine planted in 1956 | NW | 760 – 1021 | 13 | *Pinus strobus* |
| Clear Cut | WS36 | Undisturbed since 1923 | ESE | 1021 – 1542 | 49 | *Rhododendron maximum, Liriodendron tulipifera, Betula spp.* |
|  | WS37 | All woody vegetation cut in 1963, no products removed | ENE | 1033 – 1592 | 44 | *Liriodendron tulipifera, Betula spp.* |

Additional watershed information can be found at: <https://coweeta.uga.edu/Watersheds.html>

Table S2: Soil physicochemical variables aggregated across disturbed and reference soil samples. Means ± one SE shown. Asterisks indicate significantly higher values (mixed effects models) at the following significance levels: * *P* < 0.05, ** *P* < 0.01, *** *P* < 0.001.

| Variable | Reference | Disturbed |
| --- | --- | --- |
| pH | 5.25 (0.038) | 5.56 (0.052)*** |
| Moisture (g H_2_O g soil^-1^) | 0.30 (0.014) | 0.294 (0.008) |
| NO_3_ (µg N g soil^-1^) | 0.119 (0.040) | 1.08 (0.443)*** |
| NH_4_ (µg N g soil^-1^) | 1.79 (0.140) | 2.53 (0.187)*** |
| DOC (µg C g soil^-1^) | 391.9 (13.0)*** | 302.7 (17.7) |
| TDN (µg N g soil^-1^) | 46.7 (2.41) | 42.8 (2.59) |
| DOC:TDN  DON (µg N g soil^-1^) | 8.61 (0.264)*  44.7 (2.30)* | 7.22 (0.286)  39.1 (2.50) |
| Microbial Biomass C (µg C g soil^-1^) | 204.6 (13.3)* | 176.0 (15.8) |
| Microbial Biomass N (µg N g soil^-1^)  Microbial Biomass C:N  Total C (mg C g soil^-1^) | 41.5 (2.62)  4.85 (0.174)*  41.3 (3.18) | 40.0 (3.27)  4.30 (0.115)  45.5 (4.95) |
| Total N (mg N g soil^-1^) | 2.35 (0.203) | 2.90 (0.328)* |
| C:N | 17.9 (0.440)** | 16.1 (0.354) |
| SIR (µg CO_2_-C g soil^-1^ d^-1^) | 82.8 (4.13) | 93.1 (7.52)* |


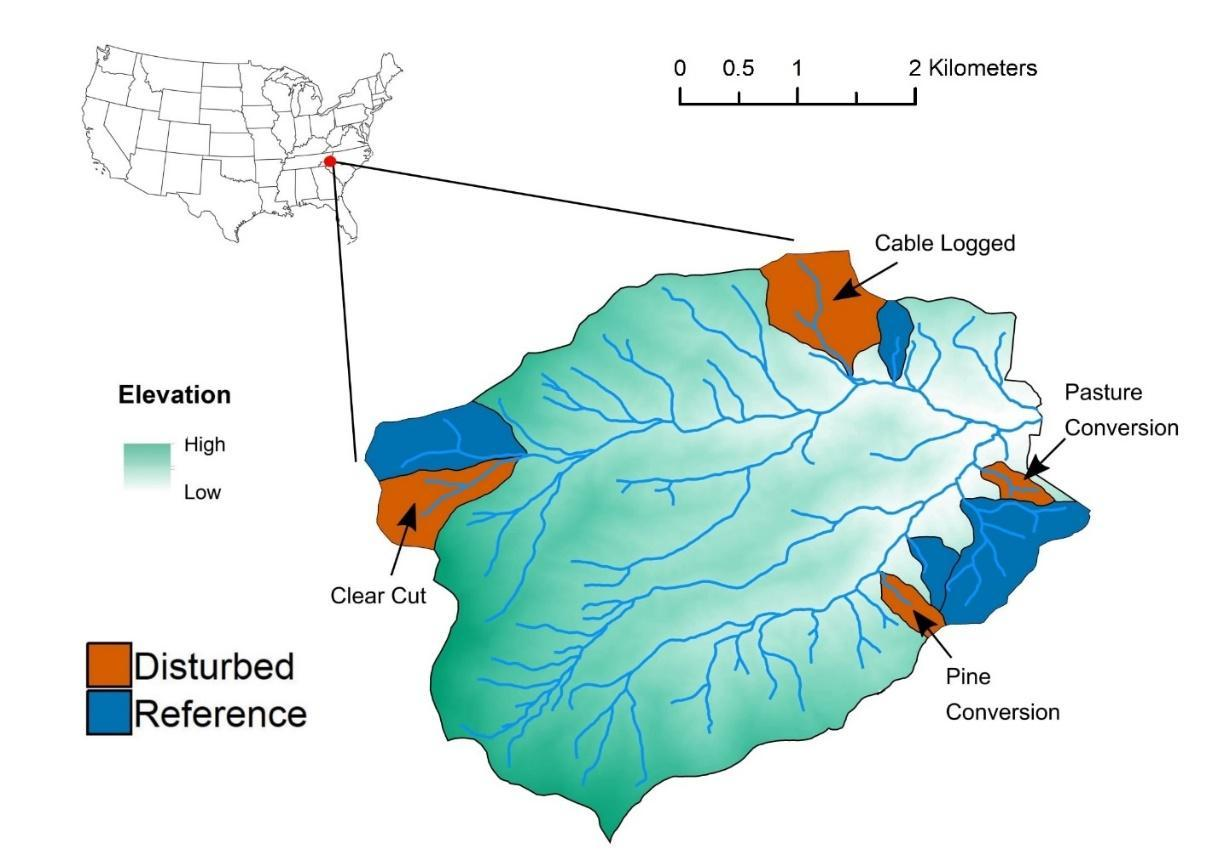


Figure S1: Map of watershed pairs at the USDA Forest Service Coweeta Hydrologic Laboratory sampled for this study


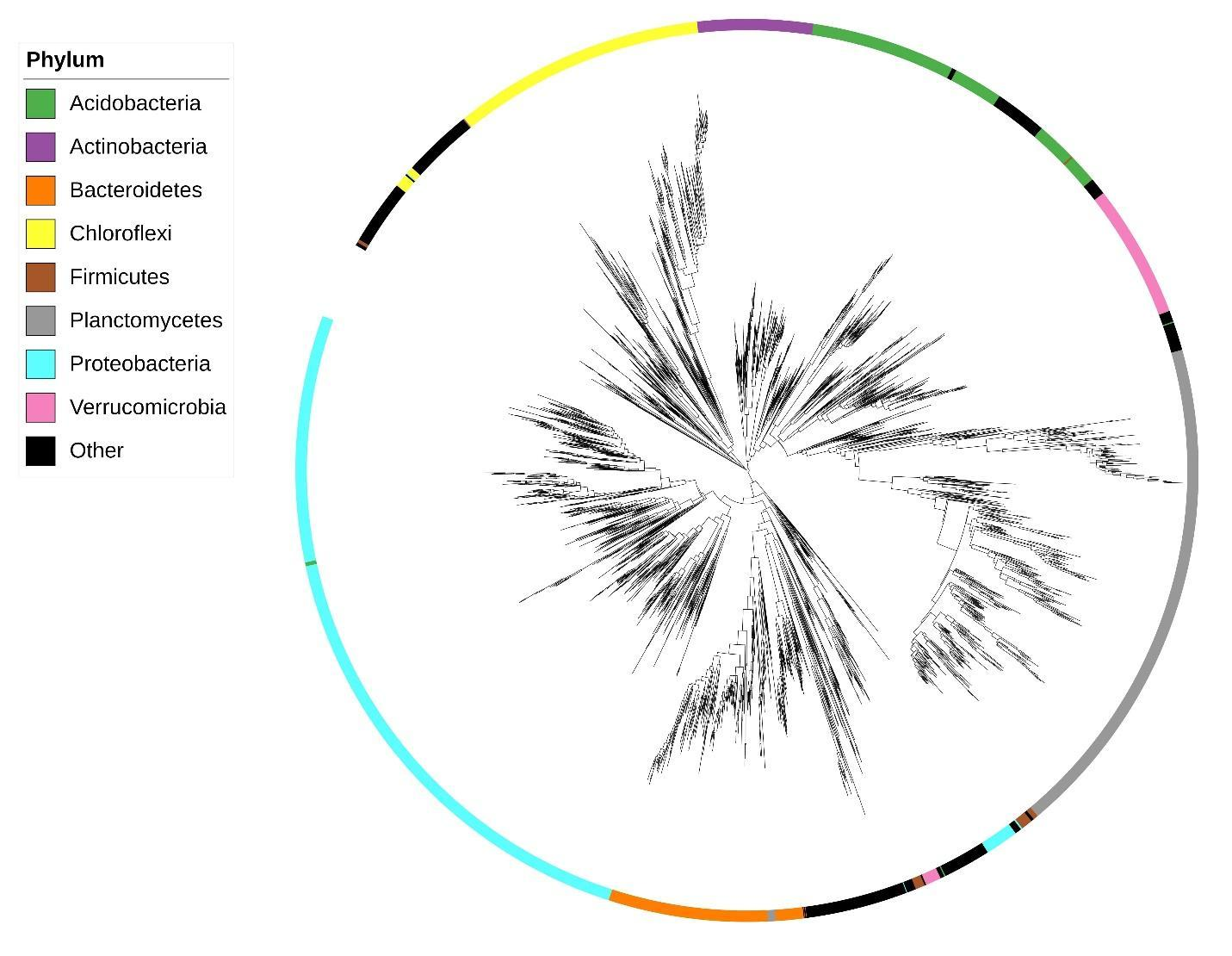


Figure S2: Maximum-likelihood bacterial phylogenetic tree inferred by FastTree2 using SILVA-aligned 16S sequences. Taxonomic assignments for 97% OTUs shown on the tree were made using a naïve-bayes classifier trained on the SILVA database. For visualization purposes, only OTUs that were successfully classified at the phylum level are shown.


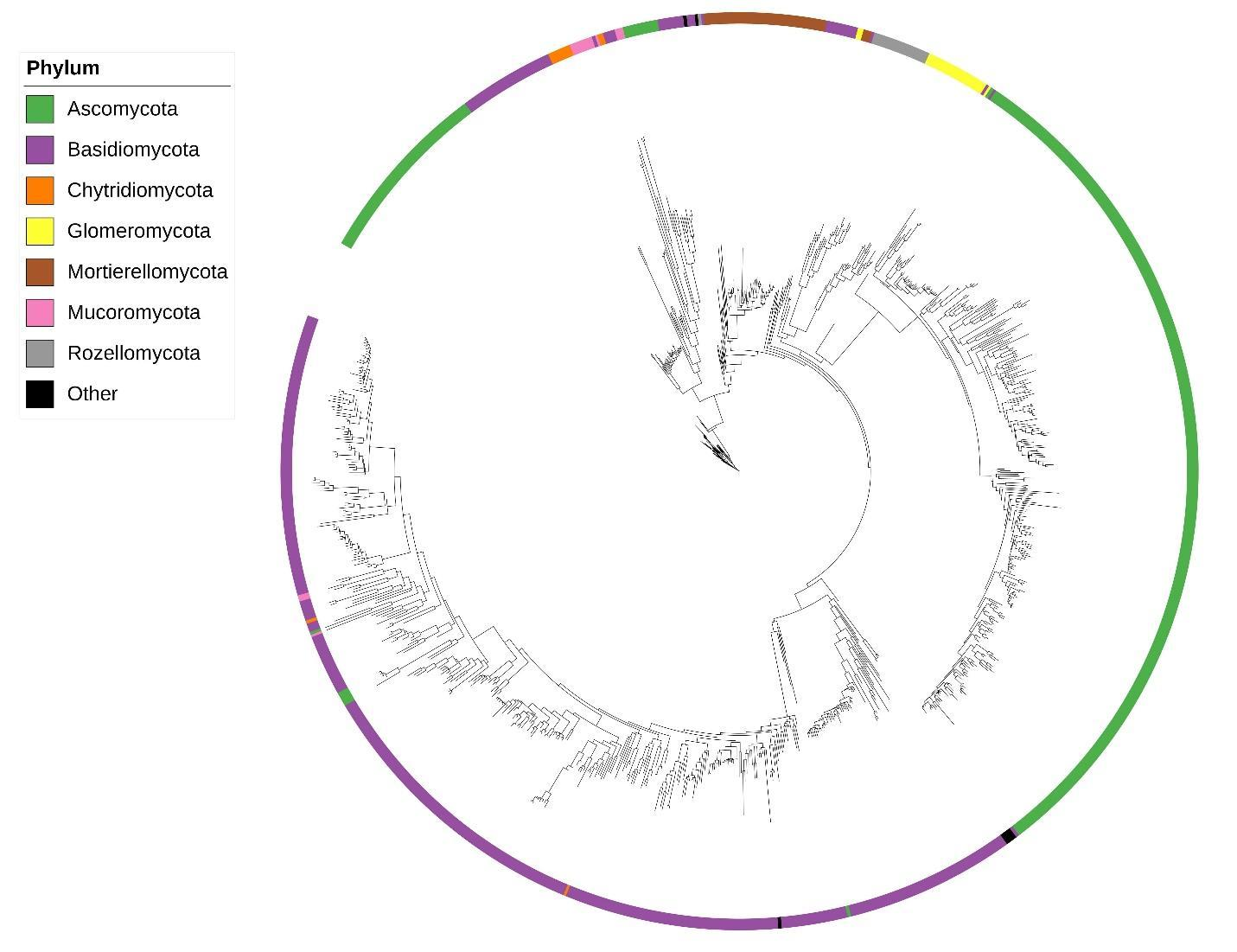


Figure S3: Maximum-likelihood fungal phylogenetic tree inferred by FastTree2 using PASTA-aligned ITS sequences. Taxonomic assignments for 97% OTUs shown on the tree were made using a naïve-bayes classifier trained on the UNITE database. For visualization purposes, only OTUs that were successfully classified at the phylum level are shown.


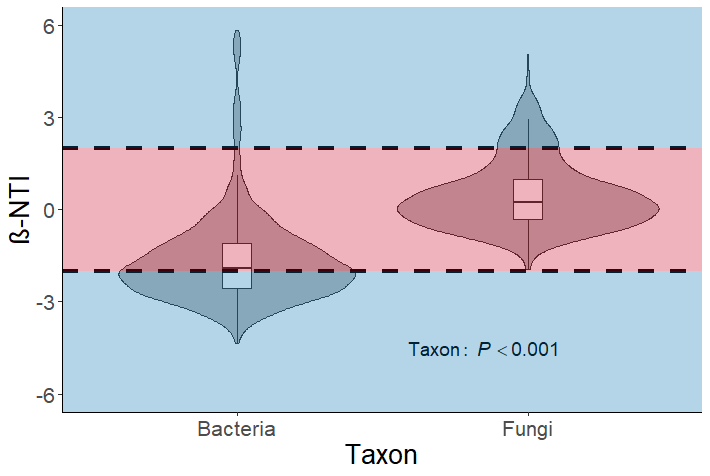


Figure S4: Bacterial and fungal βNTI distributions. *P* value is from a Kruskal-Wallis test. For this analysis, fungal phylogenetic distance was determined using a phylogenetic tree derived from UNITE taxonomy for fungal OTUs using the *taxonomy_to_tree.pl* script from Tedersoo et al. [15]. The results are nearly identical to those from the fungal maximum-likelihood FastTree using PASTA-aligned ITS sequences (Fig 1A).


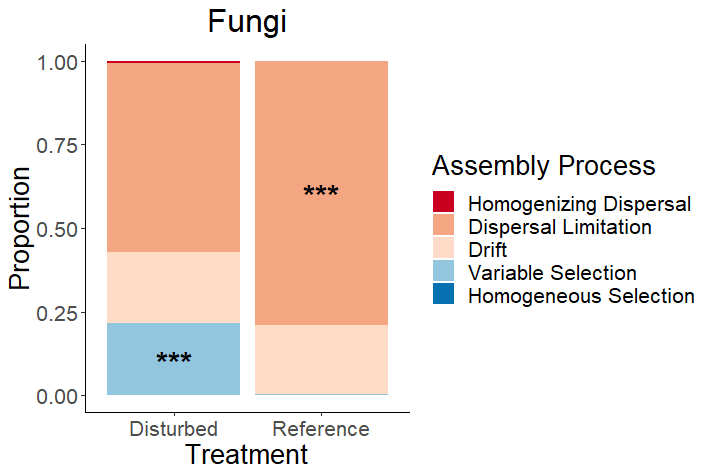


Figure S5: Proportions of specific assembly processes across land uses for fungal communities. Asterisks represent statistical significance (Z-tests) at the following significance level: *** *P* < 0.001. For this analysis, fungal phylogenetic distance was determined using a phylogenetic tree derived from UNITE taxonomy for fungal OTUs using the *taxonomy_to_tree.pl* script from Tedersoo et al. [15]. The results are nearly identical to those from the fungal maximum-likelihood FastTree using PASTA-aligned ITS sequences (Fig 1C).


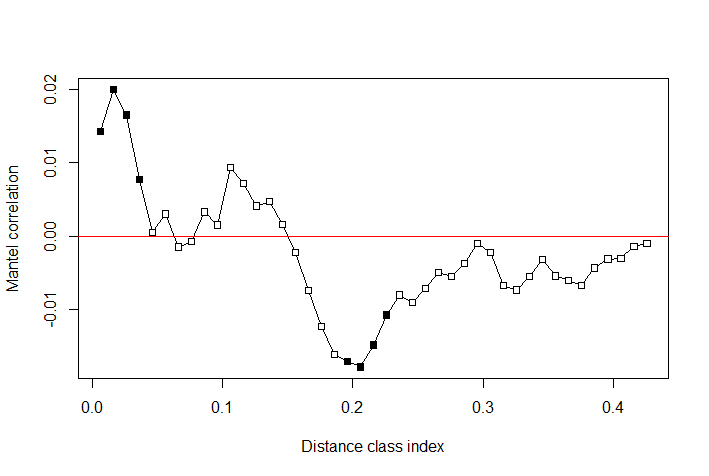


Figure S6: Mantel correlogram for pairwise Euclidean distances of bacterial OTUs’ optimal environmental conditions (calculated using scaled relative abundance-weighted means of soil properties) and phylogenetic distances (normalized between 0 and 1). Black squares indicate significant correlations (*P* < 0.05) after Bonferroni correction for multiple comparisons.


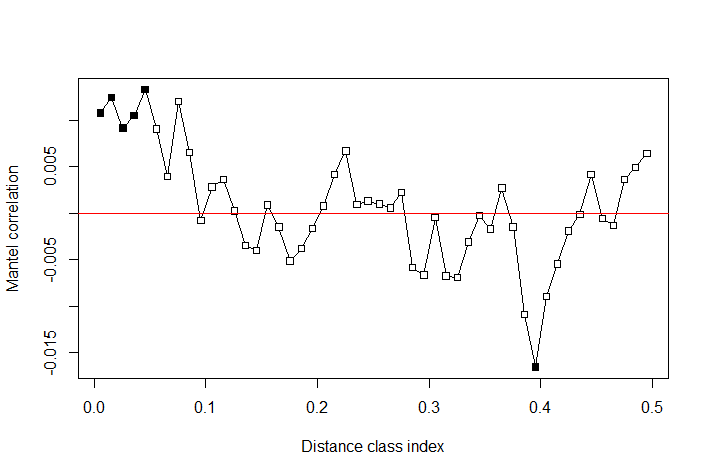


Figure S7: Mantel correlogram for pairwise Euclidean distances of fungal OTUs’ optimal environmental conditions (calculated using scaled relative abundance-weighted means of soil properties) and phylogenetic distances (normalized between 0 and 1). Black squares indicate significant correlations (*P* < 0.05) after Bonferroni correction for multiple comparisons.


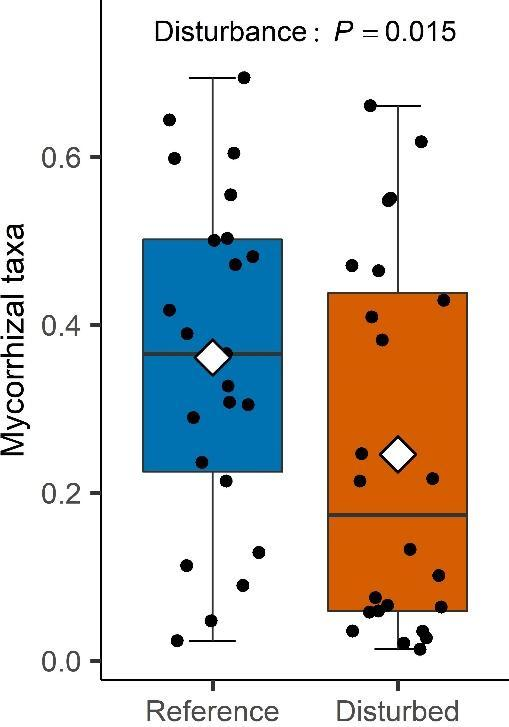


Figure S8: Relative abundances of fungal ITS sequences identified as mycorrhizal taxa by FUNGuild [22]. Data points represent the summed relative abundances of arbuscular, ericoid, orchid, and ectomycorrhizal taxa. White diamonds represent mean values for each land use. The *P* value is from a linear mixed effects model, with ‘Disturbance’ as a fixed effect and ‘Watershed Pair’ as a random effect.

**References**

1. Osburn ED, et al. Soil Bacterial and Fungal Communities Exhibit Distinct Long-Term Responses to Disturbance in Temperate Forests. *Front Microbiol* 2019; **10**.

2. Apprill A, McNally S, Parsons R, Weber L. Minor revision to V4 region SSU rRNA 806R gene primer greatly increases detection of SAR11 bacterioplankton. *Aquat Microb Ecol* 2015; **75**: 129–137.

3. Parada AE, Needham DM, Fuhrman JA. Every base matters: assessing small subunit rRNA primers for marine microbiomes with mock communities, time series and global field samples. *Environ Microbiol* 2016; **18**: 1403–1414.

4. Bellemain E, et al. ITS as an environmental DNA barcode for fungi: an in silico approach reveals potential PCR biases. *BMC Microbiol* 2010; **10**: 189.

5. Bolyen E, et al. QIIME 2: Reproducible, interactive, scalable, and extensible microbiome data science. *PeerJ* 2018.

6. Callahan BJ, et al. DADA2: High-resolution sample inference from Illumina amplicon data. *Nat Methods* 2016; **13**: 581–583.

7. Rognes T, Flouri T, Nichols B, Quince C, Mahé F. VSEARCH: a versatile open source tool for metagenomics. *PeerJ* 2016; **4**: e2584.

8. Pedregosa F, et al. Scikit-learn: Machine Learning in Python. *J Mach Learn Res* 2011; **12**: 2825–2830.

9. Abarenkov K, et al. The UNITE database for molecular identification of fungi – recent updates and future perspectives. *New Phytol* 2010; **186**: 281–285.

10. Quast C, et al. The SILVA ribosomal RNA gene database project: improved data processing and web-based tools. *Nucleic Acids Res* 2013; **41**: D590–D596.

11. Wang P, et al. Mechanisms of soil bacterial and fungal community assembly differ among and within islands. *Environmental Microbiology* 2020; **22**: 1559–1571.

12. Pruesse E, Peplies J, Glöckner FO. SINA: Accurate high-throughput multiple sequence alignment of ribosomal RNA genes. *Bioinformatics* 2012; **28**: 1823–1829.

13. Price MN, Dehal PS, Arkin AP. FastTree 2 – Approximately Maximum-Likelihood Trees for Large Alignments. *PLOS ONE* 2010; **5**: e9490.

14. Mirarab S, et al. PASTA: Ultra-Large Multiple Sequence Alignment for Nucleotide and Amino-Acid Sequences. *Journal of Computational Biology* 2014; **22**: 377–386.

15. Tedersoo L, et al. High-level classification of the Fungi and a tool for evolutionary ecological analyses. *Fungal Diversity* 2018; **90**: 135–159.

16. R Core Development Team. R: A Language and Environment for Statistical Computing. 2019. R Foundation for Statistical Computing, Vienna, Austria.

17. Ning D. iCAMP: Infer Community Assembly Mechanisms by Phylogenetic-Bin-Based Null Model Analysis. 2021.

18. Oksanen J, et al. vegan: Community Ecology Package. 2019.

19. Stegen JC, et al. Quantifying community assembly processes and identifying features that impose them. *The ISME Journal* 2013; **7**: 2069–2079.

20. Dini-Andreote F, Stegen JC, Elsas JD van, Salles JF. Disentangling mechanisms that mediate the balance between stochastic and deterministic processes in microbial succession. *PNAS* 2015; **112**: E1326–E1332.

21. Fillinger L, Hug K, Griebler, C. Selection imposed by local environmental conditions drives differences in microbial community composition across geographically distinct groundwater aquifers. *FEMS Microbiology Ecology* 2019; **95**.

22. Nguyen NH, et al. FUNGuild: An open annotation tool for parsing fungal community datasets by ecological guild. *Fungal Ecol* 2016; **20**: 241–248.
